# Improved siRNA/shRNA Functionality by Mismatched Duplex

**DOI:** 10.1371/journal.pone.0028580

**Published:** 2011-12-09

**Authors:** Haoquan Wu, Hongming Ma, Chunting Ye, Danielle Ramirez, Shuiping Chen, Jessica Montoya, Premlata Shankar, Xiaozhong A. Wang, N. Manjunath

**Affiliations:** 1 Center of Excellence in Infectious Disease Research, Department of Biomedical Sciences, Paul L. Foster School of Medicine, Texas Tech University Health Sciences Center, El Paso, Texas, United States of America; 2 Department of Biochemistry, Molecular Biology, and Cell Biology, Northwestern University, Evanston, Illinois, United States of America; UMDNJ-New Jersey Medical School, United States of America

## Abstract

siRNA (small interfering RNA) and shRNA (small hairpin RNA) are powerful and commonly used tools in biomedical research. Currently, siRNAs are generally designed as two 21 nt strands of RNA that include a 19 nt completely complementary part and a 2 nt overhang. However, since the si/shRNAs use the endogenous miRNA machinery for gene silencing and the miRNAs are generally 22 nt in length and contain multiple internal mismatches, we tested if the functionality can be increased by designing the si/shRNAs to mimic a miRNA structure. We systematically investigated the effect of single or multiple mismatches introduced in the passenger strand at different positions on siRNA functionality. Mismatches at certain positions could significantly increase the functionality of siRNAs and also, in some cases decreased the unwanted passenger strand functionality. The same strategy could also be used to design shRNAs. Finally, we showed that both si and miRNA structured oligos (siRNA with or without mismatches in the passenger strand) can repress targets in all individual Ago containing cells, suggesting that the Ago proteins do not differentiate between si/miRNA-based structure for silencing activity.

## Introduction

siRNA/shRNA is one of the most powerful and commonly used tool for biomedical research and is also being explored as therapeutic candidates for a number of diseases. The design of siRNAs has undergone tremendous improvements over the years and several algorithms have been developed to roughly predict functionality by studying large sets of siRNAs [1], [2], [3], [4], [5], [6], [7], [8], [9], [10], [11]. These algorithms commonly choose defined regions in the target to design siRNAs. Usually more than enough regions are available to design good siRNA candidates. However, in some cases, only limited target sequences are available. For example, highly conserved target regions must be used to design siRNAs for suppression of viral infections to avoid rapid emergence of escape mutants and such conserved regions are few and are often not the ideal sequences predicted by currently available algorithms for siRNA design. Thus, there is still a need to improve siRNA design.

In mammals, exogenously introduced siRNA and shRNA have to be processed by endogenous microRNA (miRNA) machinery in order to be functional. Thus, understanding miRNA biogenesis can provide insights into designing better RNAi strategies for application. miRNAs are genomically encoded and are transcribed as long primary transcripts (pri-miRNAs) that are processed by Drosha and Dicer into ∼65 nucleotides (nt) pre-miRNA and ∼22 nt mature miRNA duplex respectively [12], [13], [14], [15], [16], [17], [18], [19], [20], [21]. Generally one (guide) strand of duplex is loaded into RISC to repress target gene expression while the other (passenger) strand is discarded [22], [23], [24], [25]. siRNAs or shRNAs can be considered as analogs of intermediate products at different stages of miRNA biogenesis—siRNA representing mature miRNA duplex and shRNA representing pre or pri-miRNA. Currently, siRNAs are generally designed as two 21 nt strands of RNA that include 19 nt completely complementary sequences and 2 nt 3′ overhangs [7], [26], [27], [28]. However, the natural substrates of the miRNA loading system—the endogenously generated miRNA duplexes are typically 22 nt in length [29], [30] and have multiple internal mismatches. In this study, we find that siRNA functionality can be improved if conventional siRNA is changed to have a miRNA duplex-like structure by increasing the length to 22 nt and introducing mismatches into the duplex.

## Results

### Many conventional siRNAs was nonfunctional

**Figure 1:**
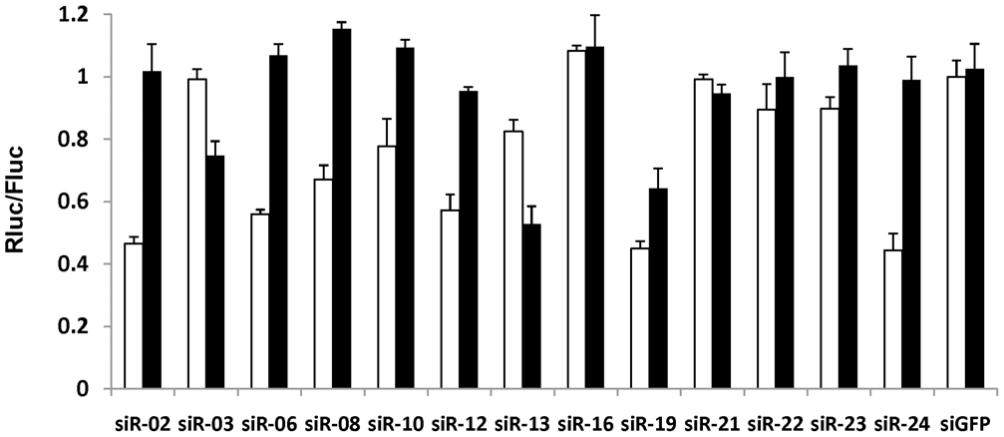
The functionality of conventional siRNAs targeting conserved regions in the flaviviral genome. Dual luciferase assay performed 24 hours after co-transfection of indicated siRNAs with the reporter vector psiCHECK2 harboring the siRNA target sequences. Target sequences of guide strand (white bars) or passenger strand (black bars) were inserted into the 3′ *UTR* of *Renilla* luciferase gene to test the intended or passenger strand functionality. The ratio of Renilla luciferase (Rluc, reporter) to firefly luciferase (Fluc, internal control), normalized to the negative control siRNA (siGFP) is shown. The experiments were performed in triplicate. Error bar = 1 S.D.

In an earlier study, we have shown that a single siRNA targeting a highly conserved region in the flaviviral genome can suppress fatal encephalitis induced by two neurotropic flaviviruses, Japanese encephalitis virus (JEV) and West Nile virus (WNV) [31]. To increase the repertoire of such siRNAs, we tested an additional 25 siRNAs targeting highly conserved regions in the viral genome [32]. Out of these 25 siRNAs, only 7 could inhibit virus infection by more than 60% (Figure 1a of reference [32]). To understand why most siRNAs did not work, we selected 13 siRNAs including some that worked and many that did not. We performed dual luciferase assays using target sequences in the Renilla luciferase 3′ *UTR*. Both strands of siRNAs were tested for functionality using luciferase reporters containing the guide or the passenger strand target sequences in the 3′ UTR. Overall, the siRNA functionality tested with luciferase assay correlated well with virus inhibition results. Although neither strand was functional in many cases where the siRNA was ineffective, in some cases such as siR-13 and siR-03, the passenger strand was more efficient than the intended guide strand in target repression (Fig. 1).

### The effect of mismatches on the functionality of siRNA

For broad-spectrum antiviral activity, siRNAs targeting highly conserved target regions that are shared between viruses need to be used. However, such shared sequences are relatively rare and this imposes restriction on target regions and sequences to be used for siRNA design. We hypothesized that designing siRNAs to mimic the miRNA structure might convert the low or nonfunctional siRNAs to potent siRNAs. In fact, most natural miRNA duplexes are 22 nt in length [29], [30] and have internal mismatches that are highly conserved across species, suggesting that these are important structural features of miRNA duplexes. Thus, we tested if increasing the length to 22 nt and introducing mismatches in the passenger strand (without changing the guide strand sequence) of siRNAs to mimic miRNA duplexes might improve the siRNA functionality. Initially, we redesigned 3 non-working siRNAs by increasing the length to 22 nt and introducing mismatches at position 1 and 12, which are generally the most unstable sites in miRNAs according to Han *et al* [17]. This was enough to increase the siRNA functionality in all 3 cases in luciferase assays (Fig. S1).

**Figure 2:**
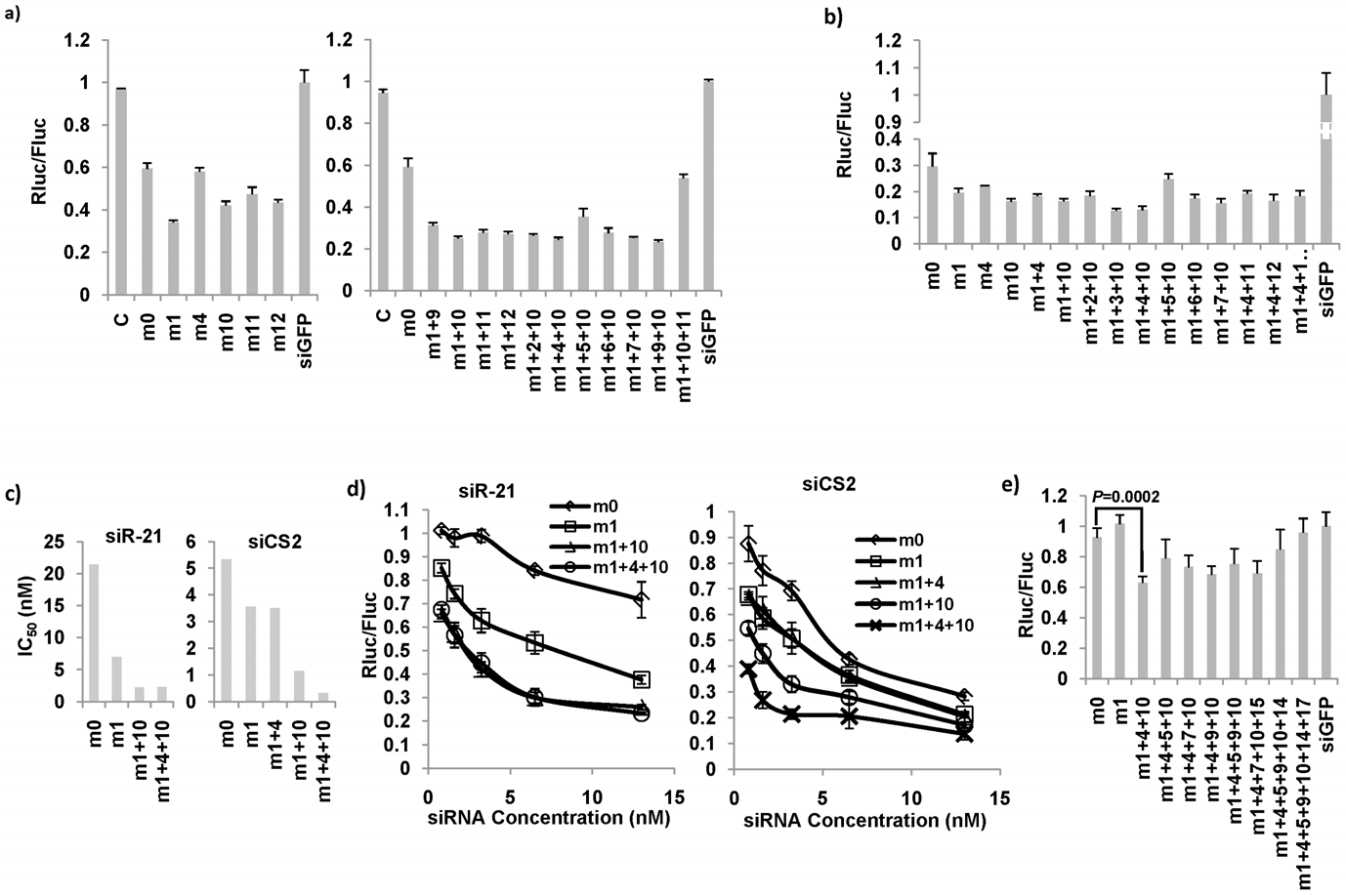
The effect of mismatches on siRNA functionality. (a) Keeping the guide strand sequence intact, single (left) or multiple (right) mismatches were introduced to the siR-21 passenger strand at the indicated positions corresponding to the guide strand 5′ end and functionality was assessed as in Fig. 1. C indicates 21 nt completely complementary siRNA, m0 represents 22 nt siRNA with no mismatch, m1 represents a single mismatch at position 1, m1+9 represent 2 mismatches at position 1 and 9 and so on. The bar graphs represent mean of triplicate. Error bar = 1 S.D. (b) Single or multiple mismatches were introduced to siCS2, a siRNA targeting highly conserved CS2 region of all mosquito-borne flaviviruses, at the indicated positions and the functionality was assessed as in Fig. 1. (c) IC_50_ was calculated for siR-21 and siCS2 with different mismatch structures based on the graph generated by testing different mismatched structures at indicated concentrations in (d). (e) Single or multiple mismatches were introduced to a siRNA targeting an artificial 100% GC sequence at the indicated positions and the functionality was assessed as in Fig. 1.

Encouraged by these results, we attempted to systematically determine the optimal mismatch structure of siRNA for efficient gene silencing. We introduced single or multiple mismatches at various positions shown in Fig. 2a to siR-21, which showed no functionality with conventional siRNA structure shown in Fig. 1. Just changing the length of siR-21 to 22 nt by replacing the 3′ dTdT with 3 nt that is perfectly complementary to the target sites without any mismatches (m0) increased the functionality of siR-21. A single mismatch in the passenger strand corresponding to guide strand position 1 (m1) or central positions, such as 10, 11, 12 increased the siRNA functionality (Fig. 2a, left). We next tested combinations of mismatches that results in the highest functionality. Introducing 2 or 3 mismatches increased the functionality further (Fig. 2a, right). To verify the results in another siRNA context, we introduced mismatches to a siRNA targeting the CS2 region of flavivirus. The CS2 region is the most conserved regions in all mosquito-borne flaviviruses and is repeated twice in the genome and thus, it is an ideal target to repress mosquito-borne flaviviruses across species. In this siRNA, a single mismatch at position 1, 4 or 10 increased the siRNA functionality, and the mismatch combinations m1+3+10 and m1+4+10 show the highest functionality (Fig. 2b).

To confirm the improved functionality of mismatched siRNAs, we determined the IC_50_ for different mismatch structures for 2 siRNAs, siR-21 and siCS2 by testing at different concentrations. As shown in Fig. 2c, the IC_50_ of mismatched siRNAs was much smaller than completely complementary siRNA for both the siRNAs tested, suggesting that mismatches improved efficacy dramatically. Examination of the siRNA titration curve shown in Fig. 2d suggests that although an improvement in functionality of mismatched siRNA was evident at each concentration tested, the improvement was much more pronounced at limiting siRNA concentrations (Fig. 2d).

It is generally believed that GC rich sequences (>50%) are not suitable for siRNA design because of high thermodynamic stability [7], [27], [33]. Because mismatches would significantly decrease the thermodynamic stability, we suspected that adding mismatches might even be able to convert GC rich sequences into efficient siRNAs. To test the hypothesis, we designed siRNAs to target an artificial sequence that consists of 100% GC. Surprisingly, m1+4+10 structure showed a small, but statistically significant repression effect while more mismatches did not increase the functionality (Fig. 2e).

The results presented above are consistent with two recent studies that showed that central mismatches facilitate RISC loading, and additional mismatches within the seed were needed to facilitate RISC maturation in Drosophila as well as mammalian cells [34], [35]. Although the above studies found that mid region mismatches promote RISC loading, we found that introducing one more mismatch in position 15 did not increase the functionality. In summary, it appears that introducing mismatches in certain positions of siRNA, such as the position 1, seed region (position 4–7), and central region (position 9–12), can increase siRNA functionality.

### Optimized structure could increase the functionality of siRNA in general

**Figure 3:**
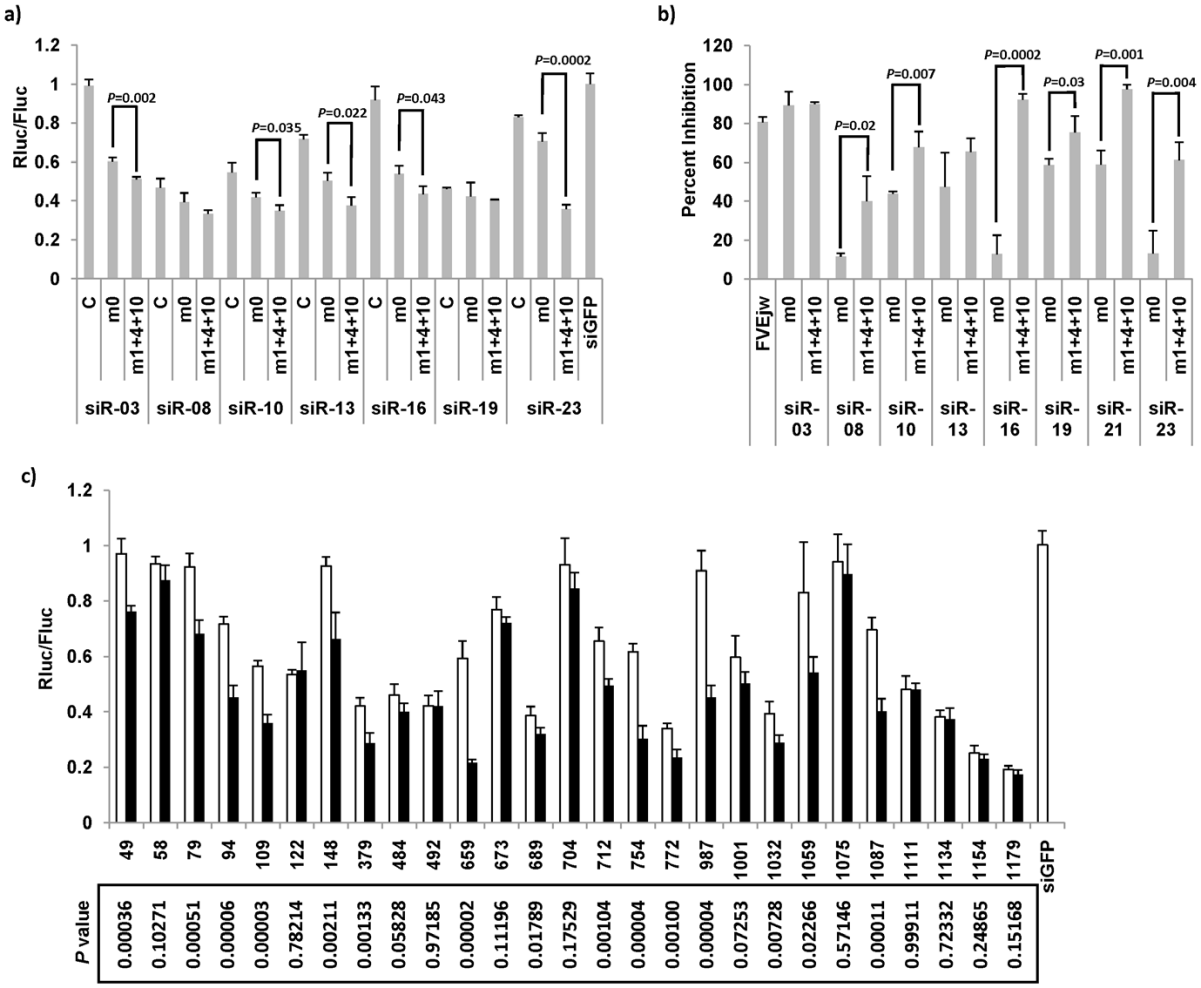
Introducing mismatches at position 1, 4 and 10 could generally increase siRNA functionality. (a) siRNAs that were not highly functional in Fig. 1 were redesigned to have 22 nt length and mismatches at position 1, 4 and 10 (m1+4+10) and tested for efficacy as in Fig. 1. (b) The redesigned siRNAs in Fig. 3a were tested for inhibition of West Nile virus replication. BHK21 cells were transfected with the indicated siRNAs and 8 h later, infected with WNV (moi = 1). Three days after infection, cells were stained with anti-West Nile Virus/Kunjin Envelope antibody and analyzed by flow cytometry to determine inhibition of virus replication. FVE^jw^ siRNA [31] was used as positive control and the data were normalized using a negative control siLuc siRNA. The bar graphs represent mean +/− SD of triplicates. (c) siRNAs targeting c-myb 3′ *UTR* were designed to have either no mismatches (m0, white) or mismatches at position 1, 4 and 10 (m1+4+10, black). The siRNA numbers represent the starting position of target sequence in c-myb 3′ *UTR*. The functionality was assessed as in Fig. 1. *P* values between m0 and m1+4+10 were shown below the figure.

Next, we tested if the optimal structure could be generalized to other siRNAs. Based on Fig. 2 results, we choose m1+4+10 as the relatively optimal siRNA structure to test the hypothesis. Seven WNV siRNAs that did not work in Fig. 1 were redesigned to have the optimal structure—22 nt in length and mismatch in the passenger strand corresponding to guide strand positions 1, 4 and 10 (m1+4+10). siRNAs without mismatches, but with the length increased to 22 nt (m0) were also included to test the effects of mismatches. In almost all cases, the redesigned siRNAs with mismatches at position 1, 4 and 10 (m1+4+10) had significantly higher functionality in the reporter luciferase assays compared to the conventional siRNAs (Fig. 3a). Consistent with previous observation with siR-21, just changing the length of siRNAs to 22 nt by replacing the 3′ dTdT with 3 nt that is perfectly complementary to the target sites without any mismatches (m0) generally increased the functionality siRNAs but introducing m1+4+10 mismatches further improved the functionality compared to m0 in 5 out of 7 siRNAs tested (Fig. 3a). The 8 redesigned siRNAs were also tested for their ability to inhibit West Nile Virus infection. Consistent with the results obtained with the luciferase assay, the redesigned siRNAs inhibited viral replication more efficiently compared to the conventional siRNAs in most cases (see Figure 1a of reference [32] and Fig. 3b). In 6 out of 8 cases, m1+4+10 showed significantly higher inhibition efficacy compared with the no mismatch 22 nt siRNAs (Fig. 3b).

Next, we randomly selected 27 targets from c-myb 3′ *UTR* with GC content ranging from 40 to 75% and designed siRNAs with no mismatch passenger strand or mismatched passenger strand at position 1, 4 and 10 (m1+4+10). The target sequences with GC content lower than 40% were purposely not selected because siRNAs with low GC content tend to disassociate when multiple mismatches are introduced. As shown in Fig. 3c, siRNAs with m1+4+10 structure showed significantly higher functionality compared to the no mismatch structure (m0) in 56% siRNAs (15 out of 27). Of note, none of the siRNAs with m1+4+10 structure showed significantly lower functionality compared with m0 structure.

In summary, introducing mismatches at position 1, 4 and 10 could increase siRNA functionality in a majority of siRNAs tested. However, it did not improve siRNA functionality in some siRNAs.

### Optimized structure can also decrease passenger strand RISC loading

**Figure 4:**
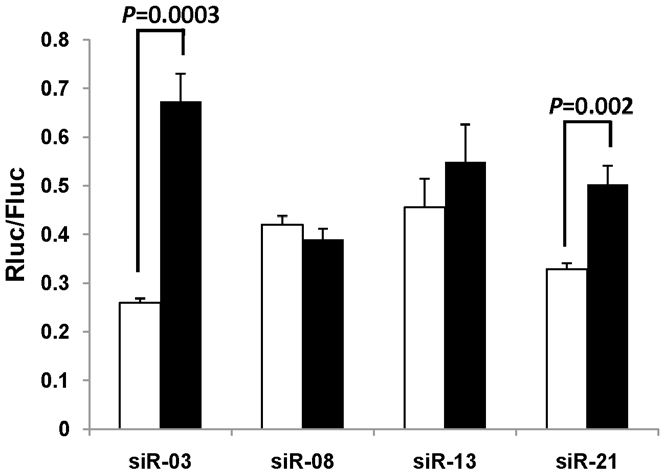
Mismatched structure could decrease the loading of passenger strand. The passenger strand functionality was compared between no mismatch (m0, white bars) and mismatched (m1+4+10, black bars) structure by co-transfecting the indicated siRNAs with psiCHECK2 vector harboring the corresponding perfectly complementary passenger strand target sequences. The ratio of Renilla luciferase (Rluc, reporter) to firefly luciferase (Fluc, internal control), normalized to the negative control siRNA (siGFP) is shown. The experiments were performed in triplicate. Error bar = 1 S.D.

One of the concerns with siRNA application is the off-target effect caused by the passenger strand loading into RISC. Often, passenger strand is also loaded [33], [36], [37], [38], [39], [40]. In this study, the passenger strand was even more efficient than the intended strand in target repression in the case of siR-03 and siR-13 (Fig. 1). It has been reported that the end thermodynamic stability dominates strand selection. Because introducing mismatches at position 1, 4, and 10 changes the thermodynamic stability of the siRNA, the loading of the passenger strand might be decreased resulting in reduced off-target effects. To test this hypothesis, we determined the passenger strand functionality in 4 siRNAs in which the functionality of the intended strand had been tested. In 2 of 4 siRNAs, the functionality of passenger strand was significantly decreased (Fig. 4). Thus, introducing mismatches to the passenger strand might reduce the off-target effect caused by passenger strand loading, at least in some cases.

### Introducing mismatches could also increase the functionality of shRNA

**Figure 5:**
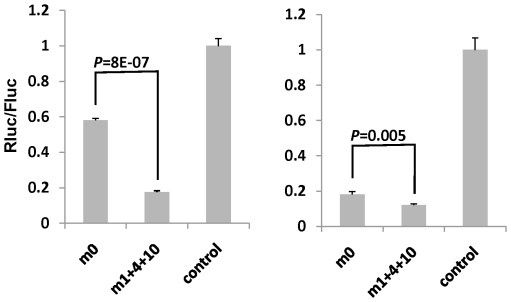
The optimal mismatch structure could also be applied to design shRNAs. shRNAs were constructed on murine pri-miR-150 backbone with no (mo) or m1+4+10 mismatches. The functionality was tested as in Fig 1. Control indicates pri-miR-150 with scrambled mature miR-150 sequence with the secondary structure kept intact. The experiments were performed in triplicate. Error bar = 1 S.D.

We hypothesized that the optimal mismatch structures might also be applied to shRNA design to enhance functionality since the shRNAs would be processed into duplexes and finally subjected to RISC loading. Currently shRNAs are commonly designed to generate perfectly complementary strands [41], [42], [43], [44], [45]. Thus, we tested if introducing m1+4+10 mismatches can enhance functionality compared to perfectly complementary shRNAs. For this purpose, we converted two siRNAs, siR-21 and a siRNA targeting the most conserved regions in HIV 5′ *UTR* [46], into shRNAs with a pri-miR-150 backbone. Compared with no mismatch structure (m0), the efficacy significantly improved with m1+4+10 mismatches introduced in the passenger strand (Fig. 5). Of note, the results with shRNA were consistent with synthetic siRNAs (Fig. 2a and Fig. S2).

### All 4 Argonaute proteins can load both si and miRNA structured oligos to generate active RISC

**Figure 6:**
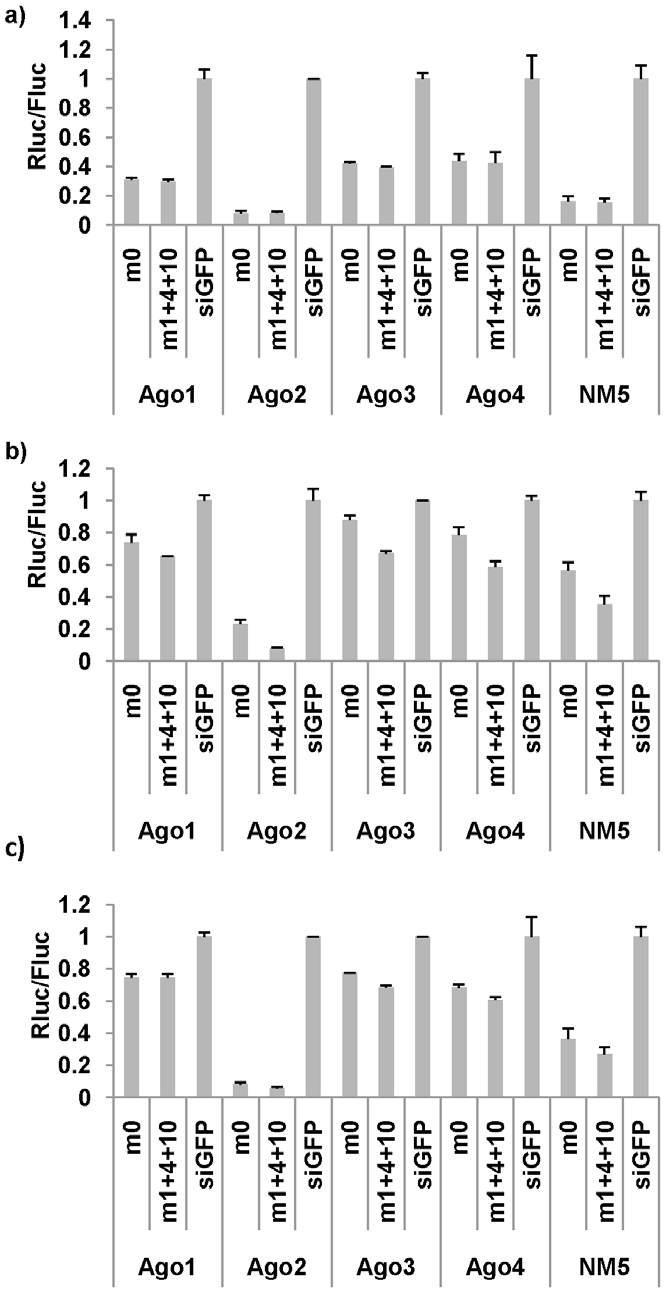
SiRNAs with and without mismatches are functional in the context of individual Ago proteins. (**a–c**) The siRNAs with or without mismatches were tested for functionality (as in Fig 1) in ES cells expressing individual Ago proteins. The functional efficacy for siRNA FVE^jw^ (a), siR-21 (b) and siR-HIV 5′ *UTR* (c) is shown. The experiments were performed in triplicate. Error bar = 1 S.D.

The four Argonaute proteins Ago1–4 constitute key components of RISC in mammalian cells [47]. In Drosophila, it has been reported that siRNAs are sorted into Ago2 while miRNAs with mismatches are sorted into Ago1 [48], [49], [50]. In mammals, such sorting does not occur and both mi and siRNAs can associate with all Ago proteins. However, a recent report suggests that while miRNA duplexes could be bound as well as unwound by all Ago proteins, siRNA duplexes could only be efficiently unwound by Ago2, although Ago1, 3 and 4 could also bind the siRNA *in vitro* [34], [35]. If this is the case, conventional siRNA should only be selected into Ago2, while miRNA-mimicking siRNAs should be able to be selected into all 4 Ago proteins, which might explain why designing siRNA to have a miRNA structure could increase the functionality. We therefore tested the repression efficacy of complementary and mismatched siRNA duplexes in mouse ES cells knocked out for all Agos followed by reconstitution with individual Agos [51] as well as in the parental NM5 ES cells expressing all 4 Ago proteins. We chose a siRNA, FVE^jw^ that did not show any functional difference when mismatches are introduced into the passenger strand. For this experiment, luciferase reporter containing completely complementary target sequences in the 3′UTR were cotransfected with FVE^jw^ siRNAs with or without mismatches in the passenger strand (thus the guide strand of both siRNAs had completely complementary target sequences in the reporter) into ES cells containing Ago1, Ago2, Ago3, Ago4 or the parental ES cells containing all Ago proteins (NM5). As expected, the Ago2 cells showed the greatest repression corresponding to its cleavage activity, whereas in Ago1, 3 and 4 cell lines, miRNA like repression accounted for the (not so profound as Ago2) reduction in target activity (with both siRNAs with or without mismatches in the passenger strand). Although the degree of repression varied between Ago2 and Ago 1, 3 and 4 cells, it is important to note that the functionality between m0 and m1+4+10 structure displayed no significant differences in Ago 1, 2, 3, or 4 cells, suggesting that all individual Ago proteins could load complementary as well as mismatched siRNA to generate active RISC similarly (Fig. 6a).

The results in another two siRNAs were consistent with FVE^jw^ results (Fig. 6b and 6c). In these two siRNAs, although mismatched siRNAs showed higher functionality in all cells, no mismatch siRNAs was clearly also functional in Ago 1, 3 and 4 cells just like in Ago2 and parental cells. Taken together, our results suggest that irrespective of the extent or mechanism of target knockdown (cleavage or miRNA like repression), both si and miRNA structured oligos (siRNA with or without mismatches in the passenger strand) are loaded into all Ago proteins and thus, the Ago proteins do not differentiate between si/miRNA-based structure for loading or maturation.

It is not surprising that the repression was more marked in Ago2 ES cells because Ago2 could cleave the target while Ago1, 3 and 4 could only repress the target by translational repression. However, it is interesting that the repression efficacy was significantly higher in Ago2 ES cells than in parental ES cells with all the Ago proteins. A recent paper reported that Ago2 was the primary rate-limiting determinant of siRNA efficacy and Ago 1, 3 and 4 could compete with Ago2 for siRNA loading [52]. This might explain the higher repression efficacy seen in Ago2 only expressing ES cells compared to the parental cell expressing all Ago proteins.

### The effect of mismatch might depend on the siRNA sequence context

**Figure 7:**
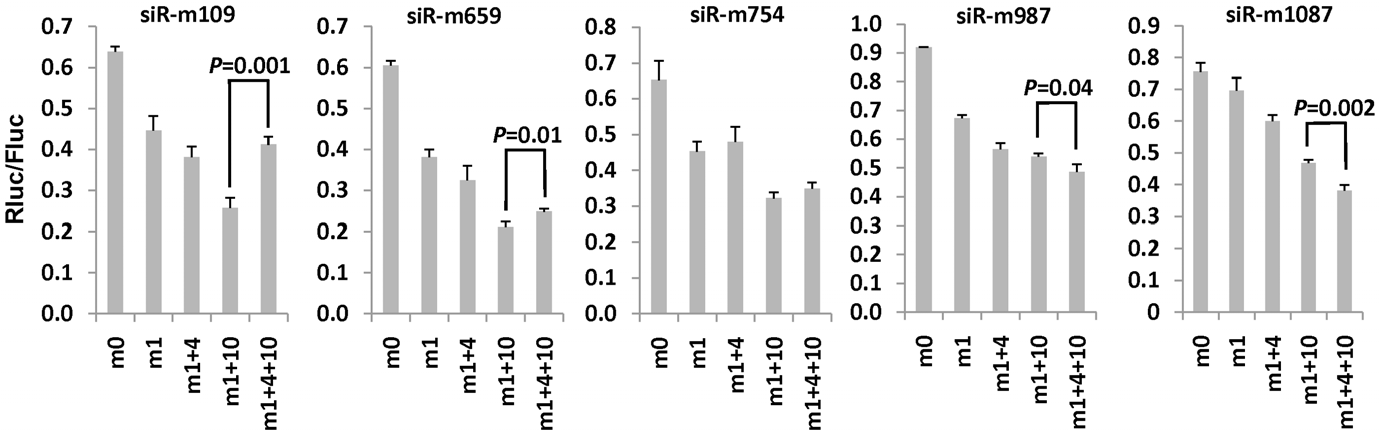
Both end and internal thermodynamic stability influence the siRNA efficacy. siRNAs targeting c-myb 3′UTR (see Fig. 3c) designed with indicated mismatches were tested for functionality as in Fig. 1.

To further dissect the contribution of mismatches at different positions in different siRNA context, we picked 5 siRNAs from the siRNAs targeting c-myb 3′ *UTR* that showed significant improvement when mismatches are introduced and compared the functionality with different mismatch structures. As shown in Fig. 7, introducing mismatch at the end (m1) or m1+4 increased the functionality to similar extent. However, introducing mismatches at more internal positions in m1+10 or m1+4+10 led to a significantly enhanced functionality for almost all the siRNAs tested, suggesting that internal thermodynamic stability strongly influences siRNA efficacy. It is noteworthy to point out that m1+10 was significantly better than m1+4+10 in siR-109 and siR-m659, suggesting that the exact optimal mismatch structure may depend on the siRNA sequence context.

## Discussion

Currently, the common practice to obtain a potent siRNA is to design several siRNAs according to the siRNA design algorithm and test their functionality. However in some cases, siRNAs have to be designed to target particular sequences. For example, highly conserved sequences shared by different virus species have to be used for broad-spectrum antiviral activity. Such conserved regions are few and are often not the ideal sequences predicted by the currently available algorithms for siRNA design. Our method provides an alternative method wherein generally any given sequence, regardless of predictability by algorithms can be optimized for gene silencing just by introducing mismatches in the passenger strand. It has been reported previously that introducing mismatch to the end of siRNA could enhance the intended strand functionality [22], [53], [54], [55]. In this study, we systematically examined the effects of introducing both end and internal mismatches and found that internal mismatches, in combination with end mismatches can significantly increase the functionality of siRNA. This method could also be applied to shRNA design.

Why does introducing mismatches improve siRNA function? Since siRNA has to be loaded into RISC to be functional, mismatches may increase the loading efficiency and thereby improve functionality. One of the major discoveries related to RISC loading of siRNA is that end thermodynamic stability dominates strand selectivity of RISC loading with the strand with less stable 5′ end showing the greatest propensity for loading [22], [53]. While this rule appears to be generally true, it is not rare that siRNAs that defy this rule are also potent in gene silencing. Moreover, siRNAs with perfect asymmetric thermodynamic ends might also not function well [56], [57]. Our study showed that in addition to the end thermodynamic stability, changing the internal thermodynamic stability by introducing mismatches at certain internal positions can further increase siRNA functionality. Kawamata *et al* reported that mismatches at the central position enhance siRNA loading into pre-RISC while mismatches at the seed region and mid region enhance siRNA unwinding [34], [35], [58]. It has also been reported that potent conventional siRNAs usually have lower internal thermodynamic stability [7], [59]. A recent paper showed that lowering the thermodynamic stability in the central position (position 9–12) by either introducing mismatches or chemical modifications, could significantly improve the siRNA potency [60]. Gu et al also showed that decreasing internal thermodynamic stability of shRNA could enhance RISC maturation by noncleaving Ago proteins, suggesting that unwinding of the 2 strands by Agos 1, 3 and 4 was enhanced by decreasing thermodynamic stability [61]. Taken together, these studies suggest that the overall thermodynamic pattern of siRNA is what really determines the siRNA functionality, not only the end thermodynamic stability. Introducing mismatches at certain positions could optimize the overall thermodynamic pattern to make the siRNA a better substrate for RISC loading and maturation.

Several previous studies have tried to improve functionality and strand-selecting accuracy of siRNAs. Chen *et al* reported that 5′ phosphate is an important determinant for strand selection and 5′-O-methylation of siRNA passenger strand could effectively reduce passenger strand functionality [38]. Several other chemical modifications have also been used to enhance functionality and decrease the selecting of passenger strand [28], [37], [55], [60], [62], [63], [64], [65].Other methods used to improve functionality and strand-selecting accuracy include incorporating 2 nt overhangs only in the guide strand, making the passenger stand into two pieces or by shortening the passenger strand by 3–4 nt [36], [39], [40]. However, none of these methods can be applied to shRNA design. Our strategy provides yet another way to enhance the functionality and strand selecting accuracy of both si and shRNAs.

It is intriguing that introducing mismatches improved functionality in some siRNAs while not in other siRNAs. Our data presented in this study could not provide a clear explanation, although some of our data might provide clues to the question. As shown in Fig. 2a, b, introducing a single mismatch at a certain position in two different siRNA appears to have different effects. For example, introducing a mismatch at position 4 showed no improvement in siR-21 compared to m0, but it had a significant functionality improvement in siCS2. Moreover, different mismatch combinations have very different effects on siR-21 and siCS2, suggesting that the effect of mismatches on siRNA functionality might be siRNA sequence-dependent. Different siRNA sequences need mismatches at different positions to improve the thermodynamic feature. Our results shown in Fig. 7a, also supports this hypothesis. It is also worth mentioning that introducing mismatches did not further improve the functionality in conventional siRNAs that already worked well (such as FVE^jw^ as shown in Fig. 6a). The reason might be that the thermodynamic pattern of these potent siRNAs might already be optimal and can't be further improved by introducing mismatches. Thus, whether introducing mismatches to improve functionality and in which position mismatches should be introduced might depend on siRNA sequences. A larger scale of study on the effect of mismatch-mediated thermodynamic pattern changes in different siRNA sequences might shed light on what is the optimal thermodynamic stability structure of siRNA for RISC loading.

How the guide strand of siRNA or miRNA is loaded into Argonaute proteins has been under intensive investigation. All 4 mammalian argonaute proteins are ubiquitously expressed and are involved in miRNA-mediated gene repression (reviewed in reference [47]). Recently, Wang *et al* reported that bacterially expressed human Ago1 and Ago2, but not Ago3 and Ago4, possess strand-dissociating activity of miRNA duplexes and passenger strand cleavage activity of siRNA duplex [66]. However, Yoda *et al* reported that although all 4 human Ago proteins showed remarkably similar structural preferences for miRNA-like duplexes, only Ago2 could load and unwind siRNA duplexes efficiently to generate mature RISC *in vitro* [35]. Our results, however suggest that the different Ago proteins do not differentiate between si/miRNA-based structure to generate mature RISC. The discrepancy between these studies might be due to the different experimental systems used. Since we performed experiments in cell lines rather than Ago proteins purified *in vitro*, our conclusion might reflect a more physiological context.

In summary, introducing mismatches in the passenger strand generally improves the efficacy of siRNA by changing the end as well as internal thermodynamic stability. Moreover this method can also be applied to shRNA design to improve efficacy.

## Materials and Methods

### Cells, oligos, transfection and luciferase assay

293FT cells (Invitrogen) were cultured according to the manufacturer's instructions. The day before transfection, 293 FT cells were trypsinized and diluted to 10^5^ cells/ml and seeded in 96 well plates in a volume of 100 µL/ well. 2 pmol siRNA and 0.1 µg psiCHECK2 plasmid harboring the target regions of testing siRNA were co-transfected with lipofectamine 2000 (Invitrogen) per the manufacturer's instructions with modifications. First, the siRNAs were mixed immediately with diluted lipofectamine 2000. Second, plasmids and siRNAs complexes were made separately. Third, the siRNAs were diluted at room temperature medium, not 37°C. Dual-Glo luciferase assays (Promega) were performed per the manufacturer's instructions one day after transfection.

To determine IC_50_, the siRNAs were serial diluted and tested for functionality. In each dilution, control siRNA duplex was added to ensure the presence of a constant amount of nucleic acid in each transfection reaction.

For shRNA functionality test, 0.1 µg shRNA construct and 0.1 µg psiCHECK2 harboring the target sequence were co-transfected into 10^4^ 293FT cells/well in a 96 well plate with lipofectamine 2000 per the manufacturer's instructions. Dual-Glo luciferase assay was performed 24 hours later.

25 conventional stabilized siRNAs targeting highly conserved region of various Flavivirus have been described [32]. All the other RNA oligos were ordered from Sigma and sequences were listed in Table S1.

### Constructs

All the DNA oligos used for constructing psiCHECK2 target reporters were listed in Table S2. DNA oligos were obtained from IDT and Sigma. The oligos were annealed and inserted into psiCHECK2 at *XhoI* and *NotI* site. Constructs were made for both sense and antisense targets separately to test strand selection. New constructs were made when mismatches were introduced into passenger strands to make sure that the target sites were perfectly complementary to the mismatched passenger strand. Full length c-myb 3′ *UTR* reporter was a kind gift from Dr. Changchun Xiao's lab.

Murine pri-miR-150 was used as the backbone for all the shRNAs. 30 nt flanking sequence at both ends of pre-miR-150 was included to ensure proper Drosha processing. The sequences of shRNA were shown in Table S3. The shRNAs were inserted into pLL3.7 vector at *HpaI* and *XhoI* site under the control of U6 promoter as described earlier [31].

### Testing siRNA for antiviral activity

The assay was performed as previously described [31]. Briefly, BHK21 cells were seeded in six well plates at 10^5^ cells per well one day before transfection. The siRNAs were transfected into cells with lipofectamine 2000 per the manufacturer's instructions. 8 hours after transfection, the cells were infected with West Nile virus (WNV) (moi = 1). 72 hours later, the cells were stained with anti-West Nile Virus/Kunjin Envelope antibody (clone 3.67G, Millipore), followed by flow cytometric analysis to determine inhibition of virus replication.

### Testing structural preference of Ago proteins

Ago1–4 expressing ES cells were cultured as described previously [51]. 2 pmol siRNA and 0.1 µg psiCHECK vectors harboring the target sequences were reverse transfected with 4×10^4^ cells per well in 96 well plates. Dual-Glo luciferase assay were performed the next day.

### Statistical analysis

Student's t test (two-tailed, assuming equal variances on all experimental data sets) was used to compare two groups of independent samples.

## Supporting Information

Figure S1 **Mismatches increase functionality.** Three siRNAs that did not effectively inhibit West Nile virus replication in our previous study (Figure 1a of reference (31)) were redesigned by increasing the length to 22 nt and introducing mismatches in the passenger strand corresponding to guide strand position 1 and 12 (m1+12) and tested for functionality as described in Fig. 1. C represents conventionally designed 21 nt siRNA with no mismatch.
(TIF)

Figure S2 **siRNA targeting highly conserved regions in the HIV 5′UTR with and without mismatches were tested for efficacy as in** **Fig. 1** **.**
(TIF)

Table S1 **The sequences of various mismatched siRNAs.** “AS” represent the guide strand while “S” represent the passenger strand.
(XLS)

Table S2 **DNA oligos used to construct psiCHECH2 vector to determine siRNA repression efficiency.** The forward and reverse strands were annealed and cloned into psiCHECK2.
(XLS)

Table S3 **shRNA sequences.** Mature duplex sequences are marked in bold. Small cap represents the mismatched nucleotides.
(XLS)
