# Supplementary figures and images for: Improved siRNA/shRNA Functionality by Mismatched Duplex
Source: PLoS One. 2011 Dec 9;6(12):e28580. doi: 10.1371/journal.pone.0028580 (PMC3235145; doi:10.1371/journal.pone.0028580)

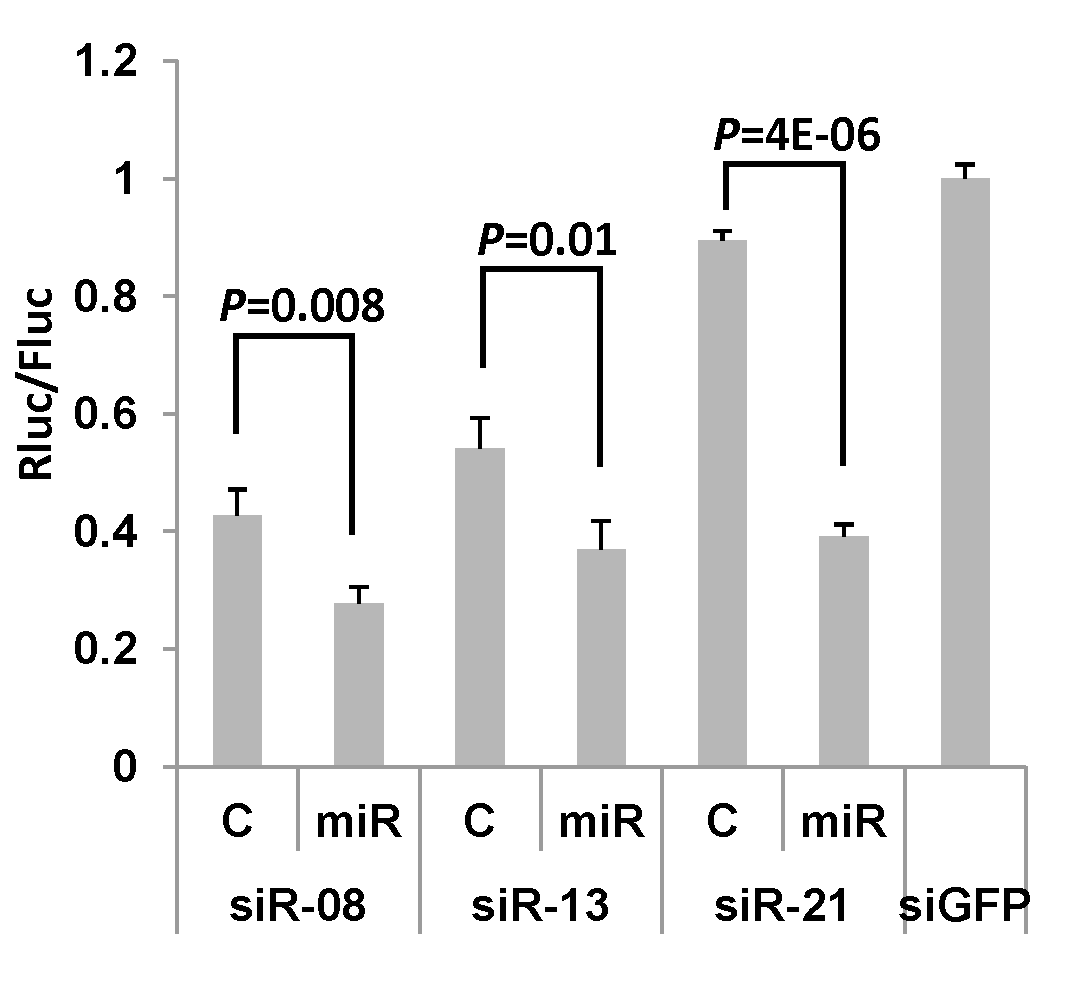

Supplement: Figure S1 — Mismatches increase functionality. Three siRNAs that did not effectively inhibit West Nile virus replication in our previous study (Figure 1a of reference (31)) were redesigned by increasing the length to 22 nt and introducing mismatches in the passenger strand corresponding to guide strand position 1 and 12 (m1+12) and tested for functionality as described in Fig. 1. C represents conventionally designed 21 nt siRNA with no mismatch. (TIF) [file pone.0028580.s001.tif]

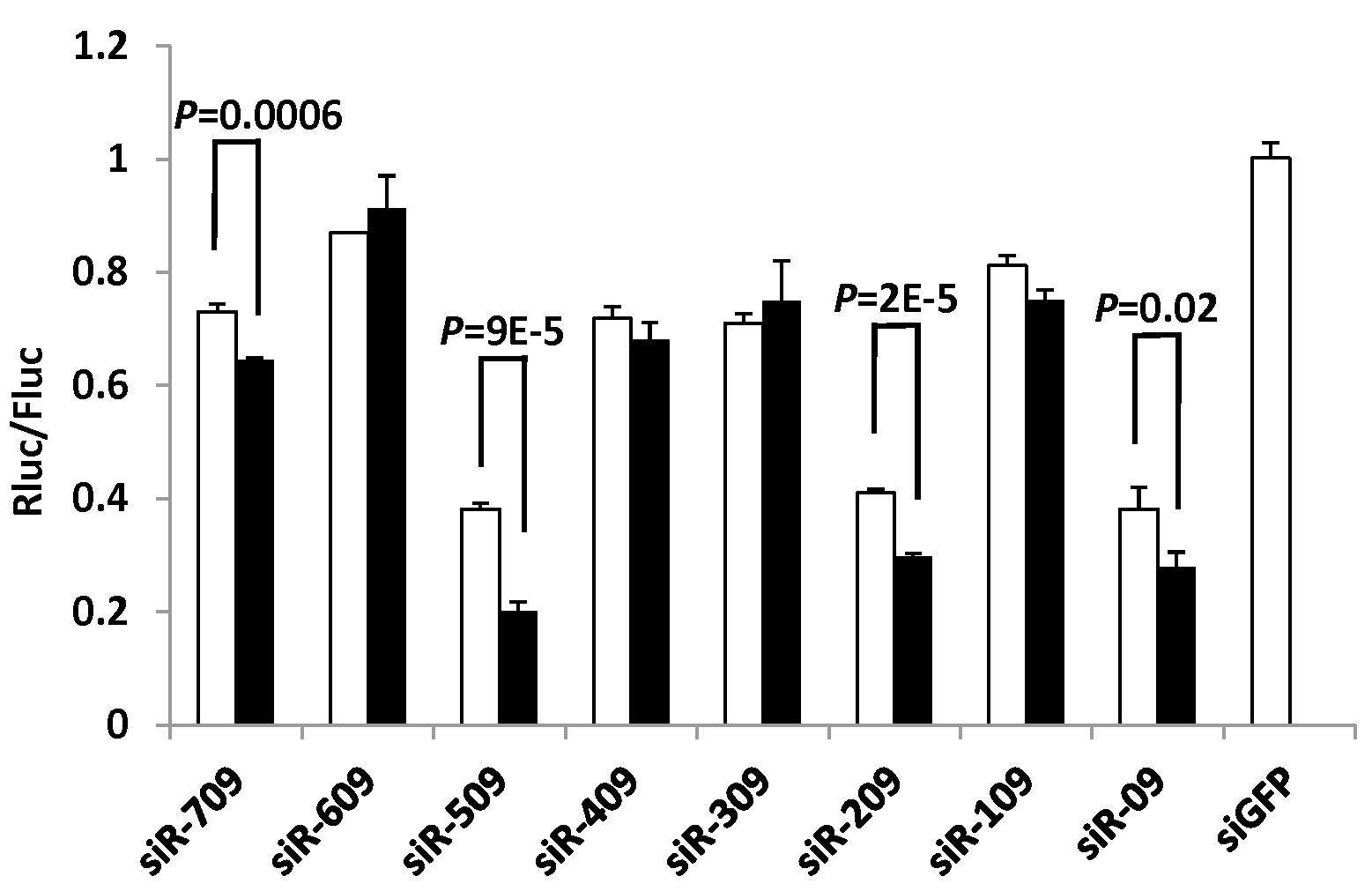

Supplement: Figure S2 — siRNA targeting highly conserved regions in the HIV 5′UTR with and without mismatches were tested for efficacy as in Fig. 1 . (TIF) [file pone.0028580.s002.tif]
